# A case series of ocular involvement in bullous pemphigoid: clinical features, management, and outcomes

**DOI:** 10.12688/f1000research.75120.1

**Published:** 2021-11-25

**Authors:** Anahita Kate, Swapna Shanbhag, Pragnya Rao Donthineni, Sayan Basu

**Affiliations:** 1 The Cornea Institute, L V Prasad Eye Institute, Vijaywada, Andhra Pradesh, India; 2 The Cornea Institute, L V Prasad Eye Institute, Hyderabad, Telangana, India; 3 Prof. Brien Holden Eye Research Centre, L V Prasad Eye Institute, Hyderabad, Telangana, India

**Keywords:** Bullous pemphigoid, cicatrizing conjunctivitis, autoimmune blistering disorders, conjunctival granuloma, case series

## Abstract

Ocular involvement in cases of bullous pemphigoid is rare and when present, the signs are usually subtle and in the form of fine tarsal scarring and dry eye disease. The current report aims to describe the clinical features and management protocols in a series of cases with aggressive ocular manifestations at presentation.

All cases of bullous pemphigoid seen between 2017 and 2020 were included in this retrospective case series. Data regarding the clinical features, treatment administered, and outcomes was collected.

Five cases (n=10 eyes) of bullous pemphigoid disease with ocular involvement were included. All eyes had significant cicatricial conjunctival changes in the form of symblephara, inferior forniceal shortening, and tarsal conjunctival scarring. Conjunctival granulomas were present in 3/10 eyes. Corneal involvement in the form of punctate keratitis was present in all eyes while 4/10 eyes had an epithelial defect as well. The management of these cases involved topical therapy with corticosteroids and lubricants (n=10 eyes) while pulse doses of intravenous methyl prednisolone were administered in 5/5 cases. Pulse intravenous cyclophosphamide was supplemented in 2/5 cases. Adequate control of the disease was noted in 3/5 cases while one case had a recalcitrant form of the disease and developed a dermalised ocular surface in both eyes. The last patient was lost to follow up during the course of therapy.

Bullous pemphigoid can present with an aggressive form of cicatrizing conjunctivitis similar to other variants of autoimmune blistering disorders and must be considered as a differential in cases presenting with ocular cicatricial disease. Long-term intensive immunosuppression is required for the management of these cases to preserve the visual function and the integrity of the globe.

## Introduction

Autoimmune blistering diseases (AIBD) are a group of disorders where the autoantibodies target antigens within the basement membrane zone (BMZ) of the skin and cause subepidermal blisters. ^1^^,^^2^ Bullous pemphigoid (BP) is the most common type of AIBD accounting for around 80% of these blistering entities. ^1^^,^^2^ The disease usually affects the elderly population (>60 years) and presents with tense, pruritic bullae that affect the flexor regions of the body. ^1^^,^^3^^,^^4^ Direct immunofluorescence (DIF) of skin biopsy samples, shows a linear deposition of C3 and IgG in the BMZ. ^1^^,^^3^ On indirect immunofluorescence, antibodies against the hemidesmosomal BP antigens 180 (BPAG2) and 230 (BPAG1) are detected in BP. ^1^^,^^3^ These findings in the context of set clinical criteria help confirm the diagnosis of BP. ^5^ The treatment involves long term systemic corticosteroids and non-steroidal agents, and the disease usually has a chronic course with frequent relapses especially on discontinuing the immunosuppressive medications.

In general, mucosal and ocular involvement is rare in BP and its absence is one of the clinical criteria for diagnosis. ^6^ Literature on ocular manifestations of BP is sparse and limited to mild conjunctival scarring, dry eye disease and rarely corneal epithelial issues. ^7^^–^^9^ The current case series aims to describe the demographic details, clinical features, treatment undergone and outcomes of five cases of bullous pemphigoid, which presented with an aggressive form of chronic cicatricial conjunctivitis.

## Case series

A retrospective review of cases with bullous pemphigoid seen between 2017 and 2020 was carried out. Written informed consent for publication and use of their records was obtained from all the patients. Data regarding the baseline demographics, duration of disease prior to presentation and the details of ongoing systemic therapy was collected. All cases underwent a detailed ocular examination that included assessment of visual acuity, slit lamp biomicroscopic examination of the lids, conjunctiva, cornea, and the rest of the anterior segment. This was followed by examination of ocular surface after staining with 2% fluorescein to document the presence of disruptions in the same. A detailed evaluation of the posterior segment was also carried out. Additionally, data pertaining to the treatment initiated at our institute along with its outcomes was collected.

A total of five cases with bullous pemphigoid were included in the current study. The baseline demographic details, details of systemic disorder, and full treatment details have been presented in Table 1.

**Table 1.** Baseline demographic characteristics and details of topical and systemic therapy administered in the cases of bullous pemphigoid.

*Case*	*Age/Gender*	*Duration of disease prior to presentation*	*Skin biopsy*	*Systemic involvement*	*Topical therapy* ^#^	*Oral immunosuppressants (duration of therapy)*	*Intravenous immunosuppressants (number of doses)*	*Surgeries undergone (number of times done)* ^*^	*Duration of follow up (months)*
Case 1	57/M	6 months	Bullous pemphigoid	Skin blisters	▪ Prednisolone acetate 1%, 8 t/d ▪ Sodium hyaluronate 0.1%, 5 t/d	▪ Prednisolone 10 mg OD (Day 0-ULFU)	IV MP 500 mg (6) IV cyclophosphamide (5)	RE: Cataract surgery+IOL	12
Case 2	36/M	2 years	Bullous pemphigoid	Skin blisters	▪ Prednisolone acetate 1%,8 t/d ▪ Chloramphenicol 1% + Hydrocortisone 0.5% ointment OD ▪ Hydroxypropyl methylcellulose 0.3%, 8 t/d	▪ Prednisolone 10 mg OD (Day 0-ULFU) ▪ Mycophenolate mofetil 500 mg BD ▪ (Day 90-ULFU)	IV MP 500 mg (8)	Nil	23
Case 3	66/F	3 weeks	Inconclusive	Skin blisters and oral ulcer	▪ Prednisolone acetate 1%, 6 t/d ▪ Chloramphenicol + Polymyxin + Dexamethasone + Sodium Phosphate + Phenyl mercuric nitrate ointment BD ▪ Carboxymethylcellulose 0.5%, 8 t/d	▪ Prednisolone 20 mg OD (Day 0-ULFU) ▪ Cyclophosphamide 50 mg OD (Day 12-ULFU) ▪ Azathioprine 50 mg OD (Day 46-ULFU)	IV MP 500 mg (3)	Amniotic membrane transplantation (2)+ tarsorrhaphy	15
Case 4	79/M	4 months	Not performed	Periocular skin blisters	▪ Prednisolone acetate 1% 8 t/d ▪ Chloramphenicol + Polymyxin + Dexamethasone + Sodium Phosphate + Phenyl mercuric nitrate ointment TID	Nil	IV MP 500 mg (3) IV cyclophosphamide (3)	Peribulbar steroid injection	4
Case 5	27M	6 months	Bullous pemphigoid	Skin blisters	▪ Prednisolone acetate 1% 10 t/d ▪ Chloramphenicol + Polymyxin + Dexamethasone + Sodium Phosphate + Phenyl mercuric nitrate ointment TID ▪ Hydropropylmethyl cellulous 0.3% gel TID	▪ Prednisolone 60 mg OD (Day 1-ULFU, maintenance 10 mg OD) ▪ Cyclophosphamide 50 mg (Day 1-ULFU)	IV MP 500 mg (3) IV hydrocortisone 100 mg (4)	RE: SLET LE: AMT + Tarsorrhaphy (2), TPG, KPro (2)	29

M: male, F: Female, IV: intravenous, MP: methyl prednisolone, ULFU: until last follow up, RE: right eye, OD: once daily, BD: twice a day (bis in die), TID: three a day (ter in die), SLET: simple limbal epithelial transplant, KPro: Keratoprosthesis, TPG: tenons patch graft.

# Prednisolone acetate was tapered slowly in all cases over 6-8 weeks.

* The number of surgeries is mentioned if performed more than once.

### Case 1

**Figure 1.**
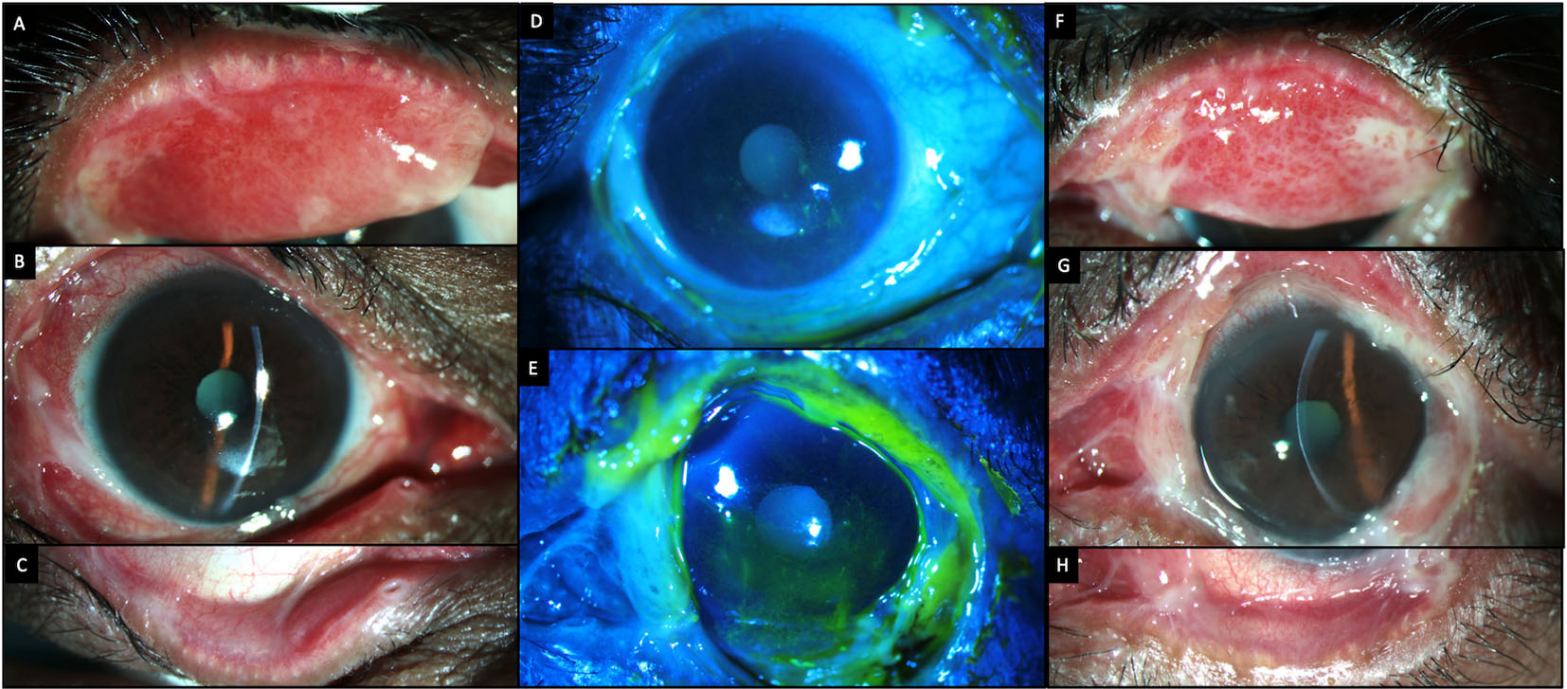
Clinical features at presentation of case 1. (A-C) Slit lamp images of the right eye showing conjunctival congestion, with symblephara laterally, inferiorly and a macular scar in the inferior cornea. Inferior forniceal shortening is also noted. (D, E) Fluorescein-stained images of the right and left eyes respectively showing punctate keratitis with filaments. (F-H) Slit lamp images of the left eye showing conjunctival congestion, with exuberant granulation tissue and cicatricial changes which are more advanced than those of the right eye.

A 57-year-old Asian-Indian man who was a businessman by profession, presented to our clinic with complaints of gradual diminution of vision with ocular pain and photophobia. He had developed skin blisters six months prior to presentation and had undergone a skin biopsy for this condition, which confirmed the diagnosis of BP. Although the patient was started on oral immunosuppressive medications (the details of which were not available) he was not compliant with the regimen. At presentation, the uncorrected visual acuity was 20/800 and 20/200 in the right and left eye, respectively. Details of ocular examination have been presented in Table 2. Slit lamp examination revealed significant conjunctival congestion with cicatricial changes in both eyes in the form of symblephara and shortening of the inferior fornices. These fibrotic changes were more pronounced in the lower bulbar and palpebral conjunctiva ( Figure 1). On eversion of the upper lids, there was active granulation tissue in both eyes. The cornea in both eyes showed superficial punctate keratitis in the central and inferior areas with filamentary keratitis.

**Table 2.** Ocular manifestations of cases of bullous pemphigoid at presentation.

	Lids	Lashes	Conjunctiva	Limbus	Cornea	Anterior chamber	Lens	Posterior segment	Schirmer test
Case 1	**BE**: inflamed, thickened lid margins. Intact mucocutaneous junction. Uninvolved puncta	**BE**: Trichiatic lashes in lower lid	**BE**: conjunctival congestion with inferior and medial symblephara and shortening of the inferior fornix. Everted upper lid: conjunctival granulation tissue	**BE**: limbal palisades-not discernable	**BE**: SPKs in central and inferior areas with filamentary keratitis. **RE**: anterior stromal macular scar abutting the visual axis inferiorly	**BE**: Quiet	**BE**: Grade three nuclear sclerosis	**BE**: WNL	**BE**: 0 mm
Case 2	**BE**: minimally inflamed lid margins. Normal mucocutaneous junction. Uninvolved puncta	**BE**: Normal	**BE**: conjunctival congestion with inferior and medial symblephara Everted upper lid: conjunctival granuloma with subconjunctival scarring	**BE**: superior and inferior limbal palisades discernable Limbal thickening+	**RE**: SPKs in central and inferior areas **LE**: Clear	**BE**: Quiet	**BE**: Clear	**BE**: WNL	NA
Case 3	**BE**: inflamed, thickened lid margins. Intact mucocutaneous junction. Uninvolved puncta	**BE**: Trichiatic lashes in both upper lids	**BE**: conjunctival congestion, shortening of inferior fornix. Everted upper lid: subconjunctival scarring	**BE**: limbal palisades-not discernable	**RE**: SPKs in central and inferior areas with two epithelial defects (3 × 1 mm) in the inferior cornea **LE**: few SPKs in the inferior cornea	**BE**: Quiet	**BE**: Pseudophakia	**BE**: WNL	**RE:** 6 mm **LE:** 0 mm
Case 4	**BE**: inflamed, thickened lid margins. Distorted mucocutaneous junction. **RE**: Uninvolved puncta **LE**: Punctal Fibrosis	**BE**: Normal	**BE**: conjunctival congestion, chemosis, inferior symblephara, shortening of inferior fornix (LE>RE). Everted upper lid: subconjunctival scarring	**BE**: limbal palisades-not discernable	**RE**: Diffuse SPKs in the inferior cornea **LE**: Diffuse SPKs with superior 7 × 4 mm epithelial defect	**BE**: Quiet	**BE**: Grade two nuclear sclerosis with posterior subcapsular cataract	**BE**: WNL	NA
Case 5	**BE**: inflamed lid margins	**BE:** Normal	**BE**: conjunctival congestion **LE**: inferior conjunctival granuloma	**BE**: limbal palisades-not discernable	**RE**: Total corneal epithelial defect with collagenolysis **LE**: Superior corneal epithelial defect (6 × 11 mm), inferior SPKs	**LE**: Quiet	**BE:** Clear	**BE**: WNL	NA

BE: Both eyes, RE: right eye, LE: left eye, SPKs: Superficial punctate keratitis, WNL: within normal limits, NA: not available.

**Figure 2.**
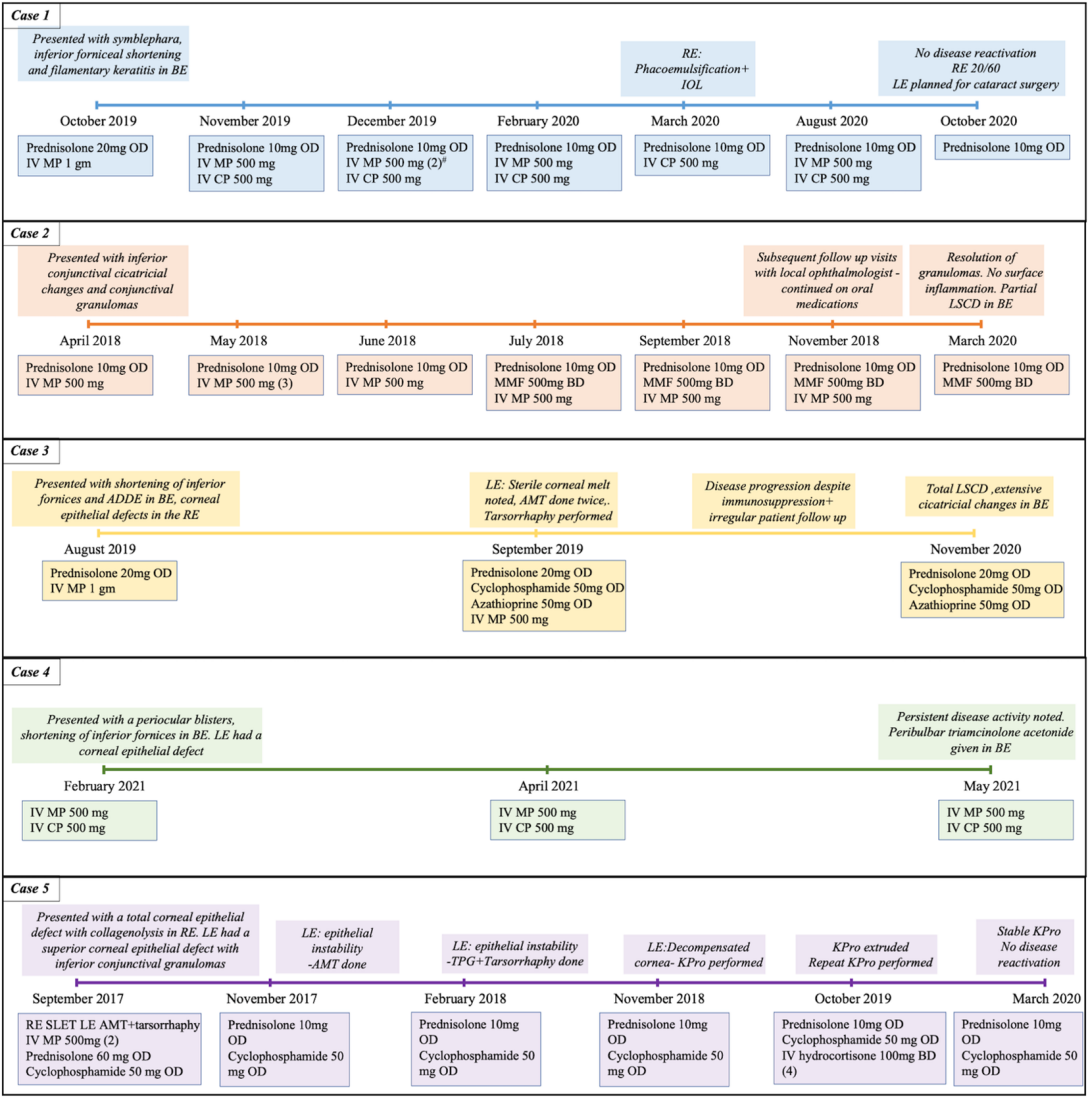
Timeline of all cases detailing the various immunosuppressive medications and interventions performed. BE: both eyes, RE: right eye, LE: left eye, IV: intravenous, MP: methyl prednisolone, CP: cyclophosphamide, TPG: tenons patch graft, AMT: amniotic membrane transplantation, SLET: simple limbal epithelial transplant, LSCD: limbal stem cell deficiency, KPro: keratoprosthesis. The numbers in brackets indicate the number of doses, if more than one was given.

The patient was started on topical therapy along with a maintenance dose of oral steroids (10 mg/day) (full information in Table 1, Figure 2). He also received three doses of intravenous methyl prednisolone (IVMP) (500 mg), given every three weeks, following which the patient was symptomatically better with improvement in surface inflammation and decrease in the granulation tissue. Five months after presentation, the patient underwent cataract surgery with an intraocular lens as there were no episodes of disease activity in the intervening period. A pulse dose IVMP (500 mg) and cyclophosphamide (500 mg) was given prior to the surgery. Postoperatively, the patient was followed up for one year with no disease reactivation noted and vision improving to 20/60. The patient has been planned for cataract surgery in the left eye in the near future.

### Case 2

**Figure 3.**
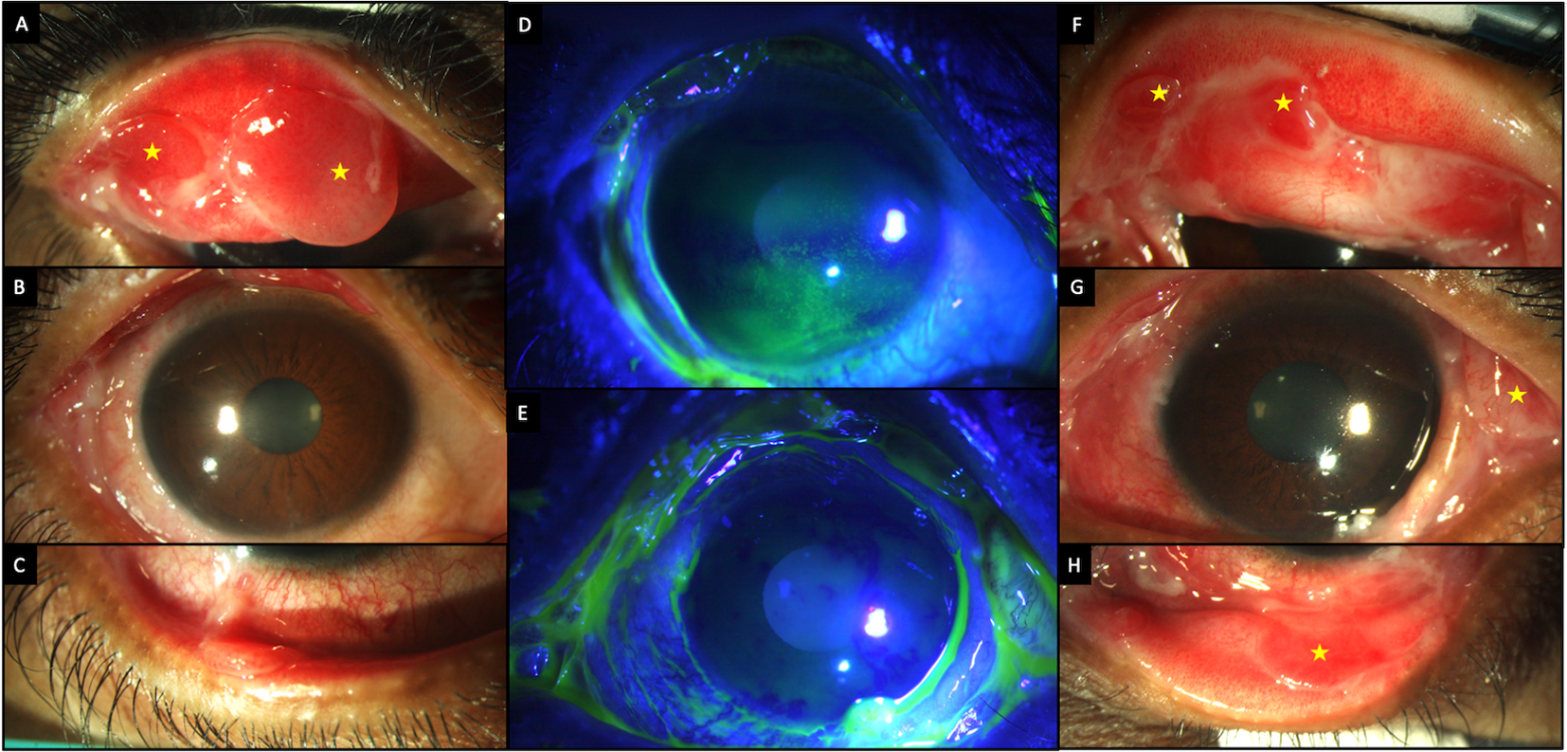
Clinical features at presentation of case 2. (A-C) Slit lamp images of the right eye showing conjunctival congestion, with two conjunctival granulomas superiorly (yellow stars) and symblephara inferiorly. (D, E) Fluorescein-stained images of the right and left eyes respectively showing punctate keratitis in the right eye. (F-H) Slit lamp images of the left eye showing conjunctival granulomas (yellow stars), with a symblepharon laterally. ^19^

A 36-year-old Asian-Indian man employed in private service, presented with pain, redness, and photophobia in both eyes for six months. He was a diagnosed case of bullous pemphigoid disease based on the results of a skin biopsy and had been prescribed oral prednisolone (10 mg/day). At presentation the corrected visual acuity in the right and left eyes was 20/25 and 20/20, respectively. The inferior conjunctiva showed significant cicatricial changes, while the eversion of the upper lid revealed conjunctival granulomas, which were larger in the right eye ( Table 2, Figure 3). The patient was given a pulse dose of IVMP (500 mg) along with topical therapy (full details in Table 1). Oral steroids (prednisolone 10 mg/day) were continued, and the patient was closely followed up.

**Figure 4.**
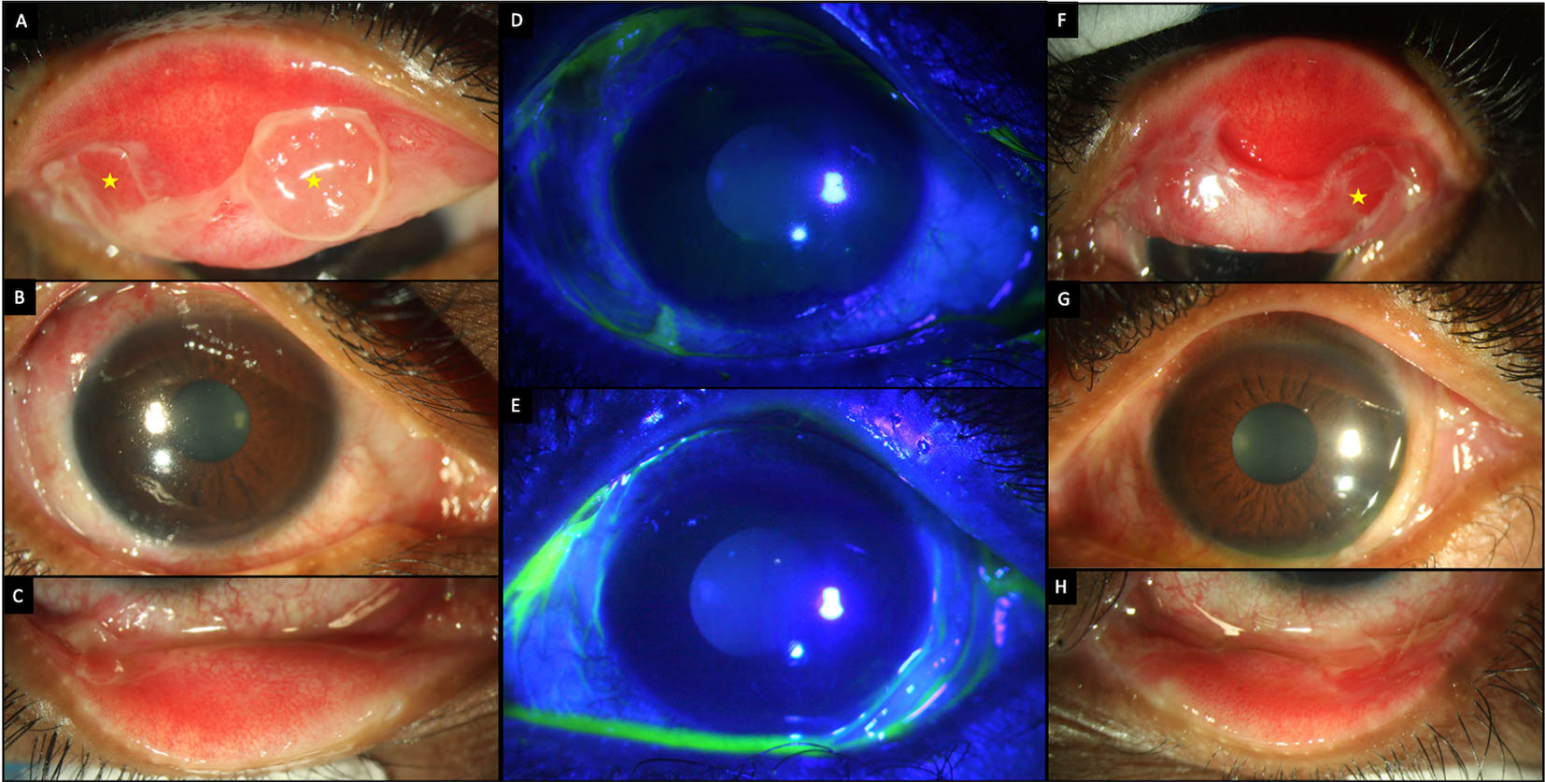
Improvement in the clinical features after two doses of pulse IV methyl prednisolone in case 2. (A-C) Slit lamp images of the right eye showing decrease in congestion with decreased size of the granulomas (yellow stars). (D, E) Fluorescein stained images of the right and left eyes respectively showing a stable surface with no corneal staining. (F-H) Images of the left eye showing decreased inflammation and smaller granulomas (yellow stars).

A significant improvement in the surface inflammation with reduction in the size of the granulomas was noted after 1 week with the topical and systemic immunosuppressants ( Figure 4). As the topical steroids were tapered, tacrolimus 0.03% eye ointment and cyclosporine 0.05% eye drops were added to the regimen. The patient continued to receive pulse IVMP (500 mg) at every follow up visit which were monthly for three months and two monthly thereafter. Oral mycophenolate mofetil was supplemented three months after presentation. No further disease exacerbation was noted and at the last follow up visit, 23 months after the initial presentation ( Figure 2) the granulomas had resolved, and the eyes had no surface inflammation. The patient maintained good vision, with a corrected visual acuity of 20/, 20/20 in the right and left eye respectively. Both eyes also had partial limbal stem cell deficiency (LSCD).

### Case 3

**Figure 5.**
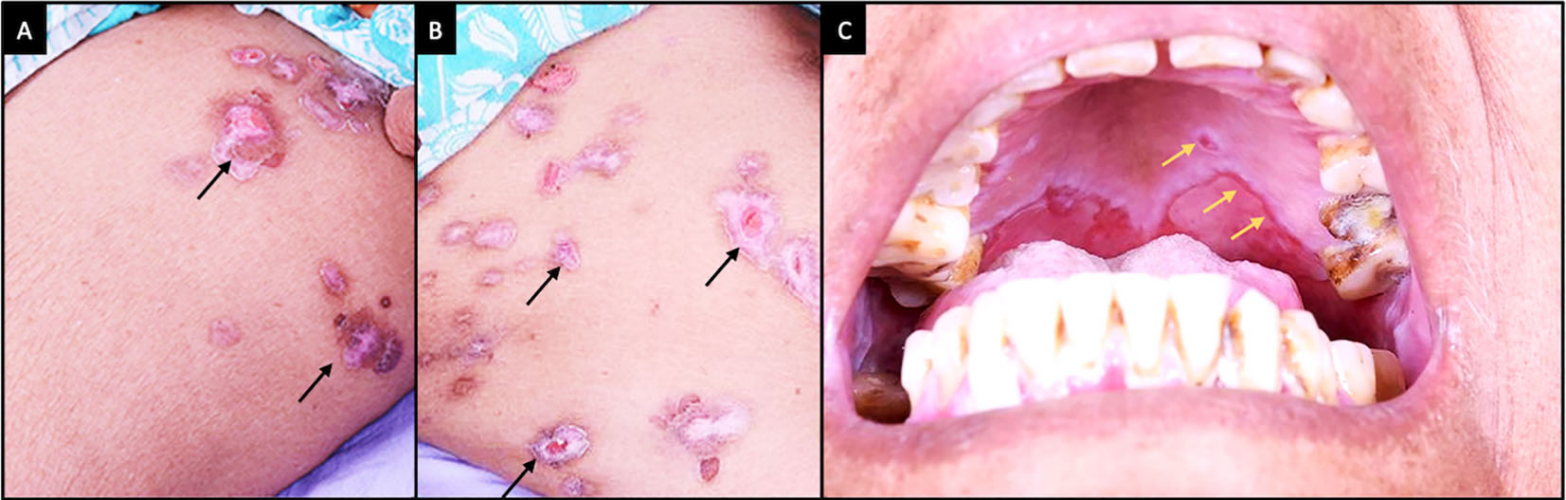
Systemic clinical features at presentation of case 3. (A, B) Ruptured blisters over the flexor aspect of the thighs (black arrows). (C) Oral ulcers over the hard and soft palate (yellow arrows).

A 66-year-old Asian-Indian woman who was a homemaker, had complaints of redness and pain in both eyes for two months. She had oral mucosal ulcers and ruptured blisters over the upper thighs and forearms during the same period ( Figure 5). The patient had previously consulted a dermatologist and was suspected to have bullous pemphigoid disease. However, the skin biopsy was not conclusive of this diagnosis. The dermatologist had started the patient on oral prednisolone, which had been tapered to 20 mg by the time of presentation to our department.

**Figure 6.**
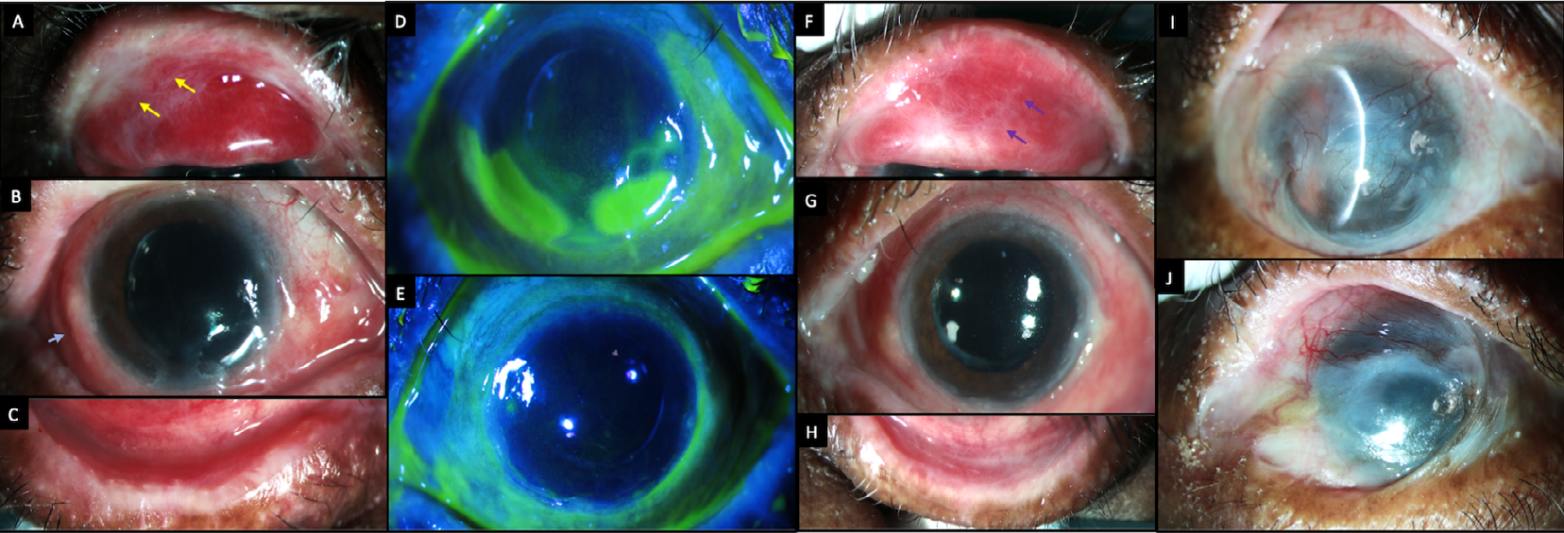
Ocular clinical features at presentation of case 3. (A-C) Slit lamp images of the right eye showing conjunctival congestion, with tarsal scarring superiorly (yellow arrows), limbal thickening (blue arrow) and inferior forniceal shortening. (D, E) Fluorescein-stained images of the right and left eyes respectively showing two epithelial defects in the inferior cornea in the right eye. (F-H) Slit lamp images of the left eye showing tarsal scarring (violet arrows) and inferior forniceal shortening. (I, J) Images of the right and left eye respectively after one year of follow up, showing total limbal stem cell deficiency with a pannus over the cornea. Left eye shows a dermalised ocular surface.

On examination, the corrected visual acuity was 20/80 and 20/50 in the right and left eye, respectively. Both eyes showed conjunctival inflammation with inferior forniceal shortening and scarring in the upper tarsal conjunctiva. The right eye had two corneal defects inferiorly ( Figure 6, Table 2). Schirmer’s test revealed aqueous deficiency (ADDE) in both eyes. A clinical diagnosis of bullous pemphigoid was made based on similarity with other cases. The patient was started on topical and oral immunosuppressants (full details in Table 1).

At the follow up visit after one month, the patient developed new skin lesions for which oral cyclophosphamide was started ( Table 1, Figure 2). A sterile corneal melt was noted in the left eye at the same visit and so the patient underwent an amniotic membrane transplantation (AMT), which was repeated after one week as the original membrane disintegrated. The patient received pulse doses of IVMP peri-operatively at weekly intervals, and oral azathioprine was also added to the regimen ( Table 1). The disease continued to progress despite the aggressive immunosuppression. Furthermore, the patient did not follow up regularly and one year after the initial presentation, the patient developed total LSCD with a vascularized, scarred cornea and extensive cicatricial changes ( Figure 6). The left eye had a dermalised ocular surface.

### Case 4

**Figure 7.**
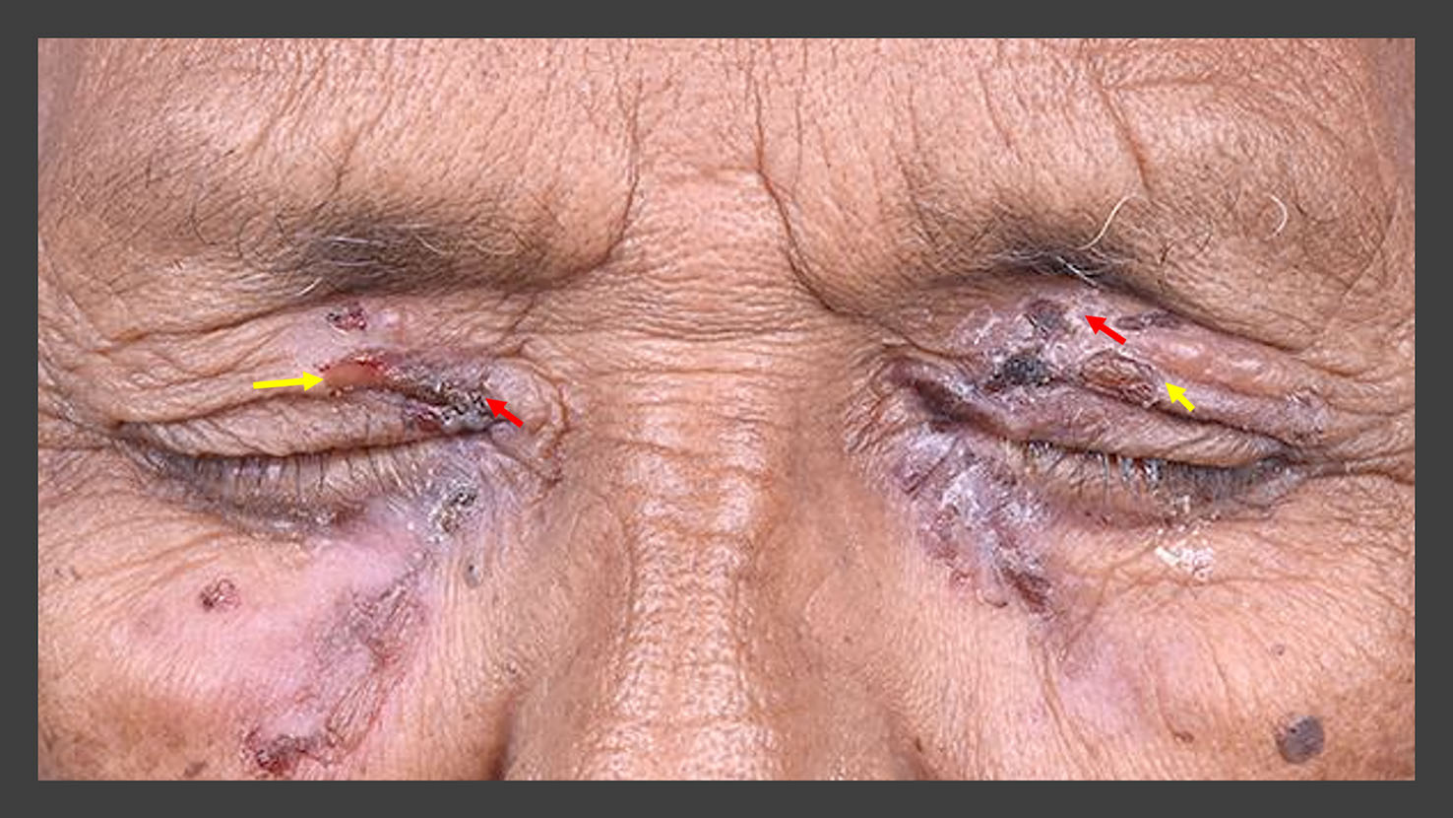
Periocular blisters around both eyes in case 4. Some of the blisters have ruptured (yellow arrows) while the others are crusted over (red arrows).

**Figure 8.**
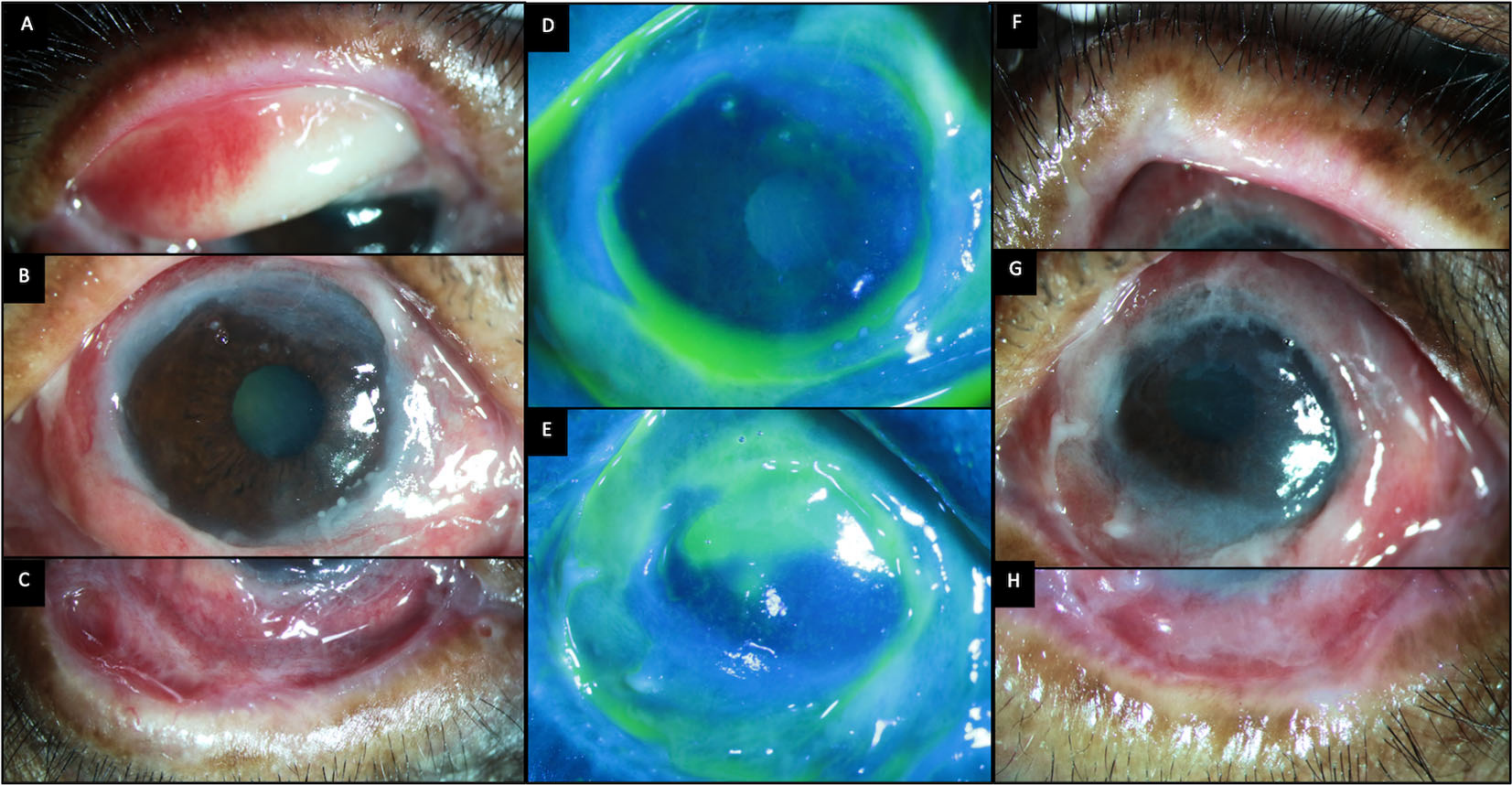
Ocular clinical features at presentation of case 4. (A-C) Slit lamp images of the right eye showing thickened distorted mucocutaneous junctions, tarsal scarring superiorly and inferior forniceal shortening with symblephara. (D, E) Fluorescein-stained images of the right and left eyes respectively showing diffuse punctate keratitis in the right eye and a superior epithelial defect in the left eye. (F-H) Images of the left eye showing thickened distorted mucocutaneous junctions and obliteration of the inferior fornix. The bulbar conjunctiva also shows thickened fibrotic changes.

A 79-year-old retired Asian-Indian man, presented with complaints of pain and redness in the eyes with discharge for four months. The patient was not on any topical or systemic therapy prior to presentation. The corrected visual acuity was 20/80 and 20/100 in the right and left eye, respectively. A periocular rash with ruptured blisters was observed in both eyes ( Figure 7). Ocular examination revealed thickened lid margins with distorted mucocutaneous junctions. However, no posterior migration was noted. The conjunctiva was inflamed in both eyes with intense cicatricial changes, especially in the inferior fornices, which showed shortening with symblephara. These changes were more pronounced in the left eye where the inferior fornix was obliterated. Both eyes had diffuse superficial punctate keratitis (SPKs) with a corneal epithelial defect in the left eye ( Table 2, Figure 8). The patient received a pulse dose of IVMP (500 mg) with IV cyclophosphamide (500 mg) along with topical medications (full details in Table 1, Figure 2). A decrease in the size of the epithelial defect was noted after 2 months, but the surface inflammation continued to persist and so the patient received another pulse dose of IVMP and cyclophosphamide. Still, a satisfactory response was not observed and so the patient received a peribulbar injection of 1ml of 4% triamcinolone acetonide in both eyes, 3 months after the initial presentation. The patient was lost to follow up after the procedure.

### Case 5

**Figure 9.**
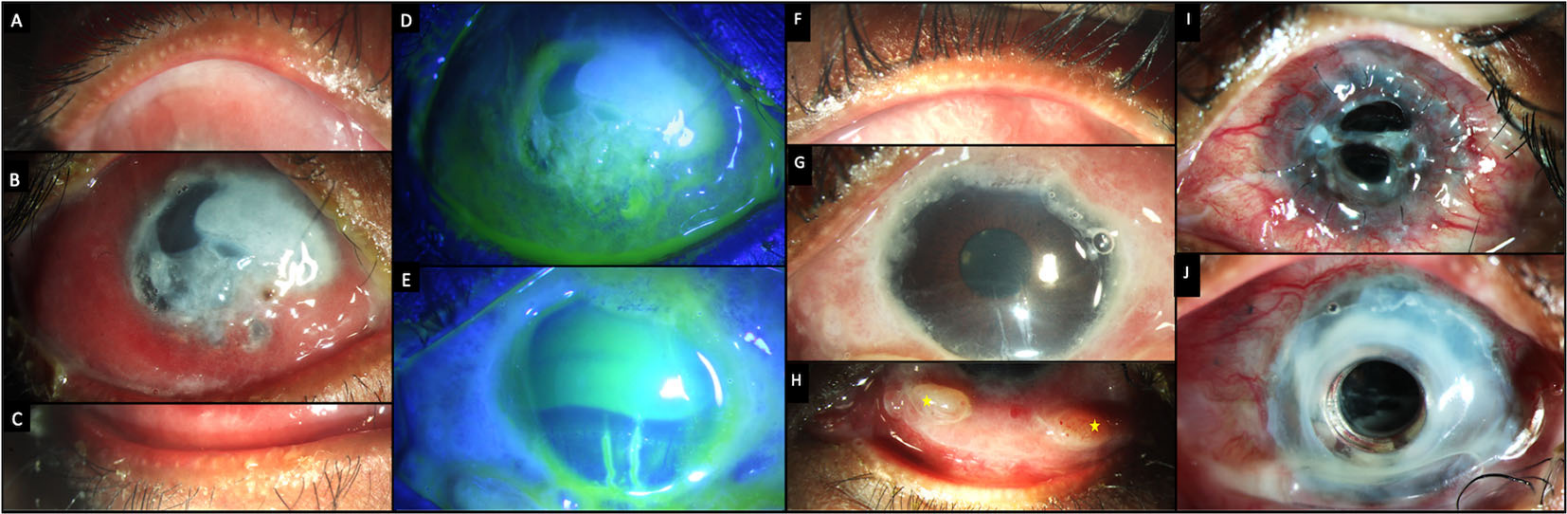
Clinical features at presentation of case 5. (A-C) Slit lamp images of the right eye showing congestion and chemosis with significant collagenolysis in the cornea. (D, E) Fluorescein-stained images of the right and left eyes respectively showing a total epithelial defect in the right eye and a superior epithelial defect in the left eye. (F-H) Images of the left eye showing intense fibrotic changes in the conjunctiva with two granulomas inferiorly (yellow stars). (I) Slit lamp image of the left eye with an extruded Keratoprosthesis. (J) Image of the same eye following a repeat keratoprosthesis, with a skirt amniotic membrane around the prosthesis. ^19^

A 27-year-old Asian-Indian man, a farmer by profession, presented with complaints of gross decrease in vision in the right eye for two weeks. He gave a history of skin blisters six months prior to presentation for which a skin biopsy was carried out and a diagnosis of bullous pemphigoid was confirmed. Systemic immunosuppressive agents were started (full details in Table 1, Figure 2). The visual acuity in the right eye was perception of light while that in the left eye was 20/50. On slit lamp examination, both eyes had significant conjunctival congestion. The right eye showed a total corneal epithelial defect with collagenolysis. The left eye had a superior corneal epithelial defect with inferior conjunctival granulomas ( Figure 9). To address the issues in both eyes, the patient underwent an allogenic simple limbal epithelial transplant from a cadaveric donor, in the right eye and an AMT in the left eye. Postoperatively, the patient was started on oral steroids and cyclophosphamide (full details in Table 1). The right eye surface stabilized over two months and was completely epithelialized with a pannus formation. The left eye continued to have epithelial instability and the patient underwent a repeat AMT and a tenons patch graft for the same. Eventually the left eye developed a decompensated cornea and a keratoprosthesis (KPro) was carried out 13 months after the initial presentation. The visual acuity improved to 20/30 and the prosthesis was stable. However, one year after the surgery, extrusion of the KPro was noted and the patient underwent a repeat KPro ( Figure 9). The second KPro was stable until the last follow up which was 4.5 months after the procedure. The patient had a vision of 20/100 in the left eye. No relapse of the systemic disease was seen, and the patient was on topical (prednisolone acetate 1%, tacrolimus 0.03% ointment) and systemic immunosuppression (prednisolone 10 mg and cyclosporine 50 mg).

## Discussion

Bullous pemphigoid disease is the most common subepidermal blistering disorder, which predominantly affects cutaneous tissue. ^3^ Though ocular involvement is rare, a few reports of early cicatricial changes exist in literature. ^4^^,^^7^^–^^9^ In the current series, all five cases presented with advanced disease and extensive conjunctival fibrotic changes, which were more severe in the inferior bulbar and palpebral conjunctiva. Corneal changes were also more localized to the inferior cornea suggesting a degree of exposure keratopathy, due to the inferior cicatricial changes, contributing to the disease process. The response to therapy was variable and usually correlated with the systemic response. This was seen in case 3, where the skin lesions continued to progress despite immunosuppression and a similar pattern of worsening of the ocular surface was also observed. This is in contrast to the first two cases where a good control of ocular inflammation was observed with no exacerbations of the systemic disease.

Although BP is considered to be a disease of the elderly, 2/5 cases in the current series presented at an earlier age. A few series have described the clinical course of BP in patients younger than 60 years and have reported a more aggressive form of the disease with higher levels of circulating autoantibodies. ^10^^,^^11^ A greater involvement of the head and neck regions has also been noted in this subgroup of population. ^10^^,^^11^ In the present series, 4/5 cases were male, which is in contrast to the female preponderance associated with BP. ^4^^,^^12^^,^^13^ A reversal of this trend of female predominance, with increased incidence of BP in males with advancing age has also been described. ^12^ Ocular findings of BP described in literature include ADDE, early tarsal scarring and pannus formation. ^7^^,^^9^ Only one case with symblephara and inferior forniceal shortening has been reported so far. ^7^ Kiyokawa *et al* reported an unusual case with corneal involvement in the absence of conjunctival involvement. ^8^ The findings in the cases described in our study represent a more aggressive form of presentation with advanced cicatricial changes and granuloma formation and have not been reported previously. The corneal involvement seen in the present series could be secondary to the inflammatory mediators over the ocular surface, the ADDE, the irregular ocular surface itself or due to the LSCD that developed over the course of the disease.

Severe, progressive fibrotic conjunctival changes with concurrent LSCD and ADDE are more commonly associated with mucous membrane pemphigoid (MMP). Traditionally patients with MMP have active or scarred lesions in the oral and nasopharyngeal mucosa with occasional cutaneous involvement. ^6^ This contrasts with BP, wherein the mucosal involvement is infrequent, and patients typically present with tense, pruritic skin blisters. Differentiating the two entities in the absence of these classical systemic findings poses a challenge because the findings on DIF for both entities are similar with linear antibody deposits in the BMZ. ^6^^,^^14^ Therefore, in cases of isolated ocular involvement further immunopathologic workup such as DIF studies on salt-split skin, enzyme-linked immunosorbent assay (ELISA) for specific autoantibodies, etc. may be required to distinguish the two blistering disorders. ^6^^,^^14^The clinical features of anti-epiligrin variant of MMP closely mimic those of case 3 presented in this report, can be considered a differential in such cases. The presence of antibodies against laminin 5 in patients sera detected by Western Blot and immunoprecipitation tests are used to confirm the diagnosis of this entity. ^15^

Systemic immunosuppressive therapy forms the mainstay of therapy in cases of BP, however a few cases of management with local immunosuppressive agents have also been reported. ^3^^,^^14^ Treatment of localized cutaneous involvement with potent topical steroids such as clobetasol propionate has shown good results. ^1^^,^^3^ A similar regimen can be adopted in isolated ocular involvement with topical or depot injections of potent corticosteroids as seen in case 4 of our series. In cases with widespread involvement administration of systemic corticosteroids is recommended. This can be given orally or in a pulsed intravenous manner. ^14^ The advantages of the latter include a faster response rate with fewer long term side effects. ^14^^,^^16^^,^^17^ All patients in the current series received a pulse therapy of IVMP either in isolation or in combination with cyclophosphamide. A maintenance dose of oral prednisolone was also a part of therapy in 4/5 cases. Several steroid sparing immunosuppressive agents such as azathioprine, mycophenolate mofetil, cyclophosphamide have been studied in BP, and have comparable response rates. ^18^^-^^20^ These agents are usually added when adequate response with the maintenance steroid dose is not observed or when an increased need for pulse doses is required. Other modalities of therapy that have been used in the management of BP include dapsone, tetracyclines, biologics, immunoglobulins, plasmapheresis, etc. and these maybe effective in recalcitrant cases. ^1^^,^^14^

In conclusion, ocular involvement in cases of BP is rare and usually subtle. In the current study, the patients presented with an advanced form of the disease characterized by severe cicatricial and granulomatous changes of the conjunctiva with LSCD, ADDE and corneal epithelial instability. Diagnosis of BP is based on a combination of classical skin blisters and a confirmatory immunofluorescence on skin biopsy. In the absence of these cutaneous findings, MMP is a close differential and additional investigations maybe required to differentiate the two diseases. Long term immunosuppression is required in the management of BP and the use of pulse IVMP with systemic steroid sparing immunosuppressive agents is usually associated with good response rates. Aggressive immunosuppression is required in cases that present with advanced ocular findings in order to preserve the visual function and to retard the chronic sequelae.

## Data availability

All data underlying the results are available as part of the article and no additional source data is required.

## Consent

Written informed consent was obtained from all the patients for publication of this case report and accompanying images.
