# Supplementary material for: A case series of ocular involvement in bullous pemphigoid: clinical features, management, and outcomes
Source: F1000Res. 2022 Jan 31;10:1201. Originally published 2021 Nov 25. [Version 2] doi: 10.12688/f1000research.75120.2 (PMC8787558; doi:10.12688/f1000research.75120.2)
Supplement: Peer review history

# Peer review: Reviewer response for version 1

**DOI:** 10.5256/f1000research.78945.r101150

**Published:** 2022-01-24

**Authors:** Jaime D. Martinez

**Affiliations:** 1 Department of Ophthalmology, Bascom Palmer Eye Institute, University of Miami Miller School of Medicine, Miami, FL, USA

Excellent review of cases. Pemphigoid is a complex disease to treat, and the importance of ocular surface details needs to be emphasized like in this article. 

**Comments:**

 1. Change the word  "non steroidal" to "anti-inflammatory or immunosuppression" Introduction first paragraph.

 2. Case 2 last paragraph  missing number 20 on 20/20

 3. Can you add details on how the patient responded to triamcinolone injection on case 4.

Are enough details provided of any physical examination and diagnostic tests, treatment given and outcomes?

Yes

Is the background of the cases’ history and progression described in sufficient detail?

Yes

Is sufficient discussion included of the importance of the findings and their relevance to future understanding of disease processes, diagnosis or treatment?

Yes

Is the conclusion balanced and justified on the basis of the findings?

Yes

Reviewer Expertise:

Ocular surfaces disease

I confirm that I have read this submission and believe that I have an appropriate level of expertise to confirm that it is of an acceptable scientific standard.

# Response

**Published:** 2022-01-27

**Authors:** Sayan Basu

**Affiliations:** L V Prasad Eye Institute, Hyderabad, India

We would like to thank the reviewer for reading and commenting on our submission. The following comments have been addressed in the manuscript. 

 1. Change the word "non steroidal" to "anti-inflammatory or immunosuppression" Introduction first paragraph.

 The required change has been made

 2.Case 2 last paragraph missing number 20 on 20/20

 Thank you for pointing this out. We have corrected the typographical error.

 3.Can you add details on how the patient responded to triamcinolone injection on case 4

 The patient unfortunately did not follow up after the triamcinolone injection and so the response to therapy is not known

# Peer review: Reviewer response for version 1

**DOI:** 10.5256/f1000research.78945.r101151

**Published:** 2022-01-13

**Authors:** Christine Shieh

**Affiliations:** 1 Department of Ophthalmology and Visual Sciences, Vanderbilt Eye Institute, Nashville, TN, USA

**Summary of the Paper**

 The authors present a case series of 5 patients with advanced ocular involving bullous pemphigoid (BP), a rare and sparsely characterized entity.  The goal was to describe the ocular features of these patients, treatment course, and outcomes of these patients.

 All patients were treated with systemic immunosuppression and steroid-sparing immunomodulatory therapy. All patients but one (case 4) had at least 1 year follow-up. Case 1 & 2 are examples of advanced ocular involving patients with good clinical outcomes with systemic immunosuppressive therapy.

 Cases 3-5 had poorer outcomes and required more invasive interventions. Case 3 developed corneal melt refractory to medical therapy and required two amniotic membrane transplants. Her course was also complicated by irregular clinical follow-up which likely impacted her clinical course. At 1 year, she had complete limbal stem cell deficiency.

 Case 4 had peribulbar triamcinolone administered but was lost to follow-up subsequently. The clinical outcome from this case is unknown at this time. Case 5 required an allogenic limbal transplant OD and amniotic membrane OS. While the right eye stabilized, the left eye endured a complicated course requiring repeat amniotic membrane, followed by K-Pro which was complicated by extrusion and required a repeat KPro.

 The case series accomplished its goal of presenting a variety of advanced cases with ocular involving BP, showcasing different clinical outcomes. Given that there are few reports of ocular involvement and most describe just early cicatricial changes, this series is very valuable for clinicians in understanding and treating ocular surface disease in patients with BP.

**Is the background of the cases’ history and progression described in sufficient detail?**

 Yes – Each of the cases are described in good detail and well-complemented by high-quality slit lamp photos. The history and clinical course/progression were further complemented by an organized timeline (Figure 2).

**Are enough details provided of any physical examination and diagnostic tests, treatment given, and outcomes?**

 Yes – the authors are very thorough in describing the various examination findings for each patient. There are good quality photos for each of the patients. Table 2 organizes each of the patient’s anterior segment exam findings very clearly. As for the treatment course, Figure 2 lays out a nice timeline for each of the 5 patients showing different treatment modalities and the clinical outcome.

**Is sufficient discussion included of the importance of the findings and their relevance to future understanding of disease processes, diagnosis, or treatment?**

 Yes – They do a great job of showing how ocular BP can manifest in such advanced stages. All 5 cases of advanced ocular involvement of BP required systemic immunosuppression with steroids and then steroid-sparing immunomodulators. In their discussion, they clearly state that advanced cases like these require systemic immunosuppression long-term.

 Case 3 & 5 presented with epithelial defects and had poorer outcomes/required more invasive interventions than Cases 1 & 2. Would love to have the authors include a few sentences in the discussion section about their clinical opinion as to why Cases 3 & 5 had poorer outcomes.  Presumably, this was due to the degree of limbal stem cell deficiency?

**Is the conclusion balanced and justified on the basis of the findings?**

 Yes – they make a well supported conclusion that patients require quick immunosuppressive therapy and long term maintenance therapy as well. Patients 1&2 were successfully treated this way, and the other patients required more invasive interventions beyond systemic medical therapy.

Are enough details provided of any physical examination and diagnostic tests, treatment given and outcomes?

Yes

Is the background of the cases’ history and progression described in sufficient detail?

Yes

Is sufficient discussion included of the importance of the findings and their relevance to future understanding of disease processes, diagnosis or treatment?

Yes

Is the conclusion balanced and justified on the basis of the findings?

Yes

Reviewer Expertise:

NA

I confirm that I have read this submission and believe that I have an appropriate level of expertise to confirm that it is of an acceptable scientific standard.

# Response

**Published:** 2022-01-27

**Authors:** Sayan Basu

**Affiliations:** L V Prasad Eye Institute, Hyderabad, India

*Case 3 & 5 presented with epithelial defects and had poorer outcomes/required more invasive interventions than Cases 1 & 2. Would love to have the authors include a few sentences in the discussion section about their clinical opinion as to why Cases 3 & 5 had poorer outcomes.  Presumably, this was due to the degree of limbal stem cell deficiency?*

 We thank the reviewer for their comments. The progressive disease noted in case 3 was likely due to the non-compliance to the systemic immunosuppressive therapy and the progression of the underlying systemic disease as well. In case 5 the gimbal stem cell deficiency aggravated the epithelial instability and hampered the healing process resulting in repeated episodes of epithelial breakdown. These points have been incorporated within the discussion
